# 198. Highway to Health: Mobile Pharmacy and Clinic Providing Hepatitis C Testing and Treatment

**DOI:** 10.1093/ofid/ofaf695.070

**Published:** 2026-01-11

**Authors:** Angela Di Paola, Sheela Shenoi, Anne E Stevens, Ralph P Brooks, Lauren Astorino, Carol A Amico, Cynthia Frank, Adati Tarfa, Sandra Ann Springer

**Affiliations:** Yale University, New Haven, Connecticut; Yale University, New Haven, Connecticut; Yale University, New Haven, Connecticut; Yale School of Medicine - AIDS Program, New Haven, Connecticut; Yale University, New Haven, Connecticut; Yale University, New Haven, Connecticut; Yale School of Medicine, New Haven, Connecticut; Yale University, New Haven, Connecticut; Yale University, New Haven, Connecticut

## Abstract

**Background:**

People who use substances face barriers when seeking treatment for hepatitis C (HCV). In 2023, the first Mobile Pharmacy and Clinic (MPC) was launched, staffed by a clinician, community health care workers, a phlebotomist, and a pharmacist. It aims to reduce barriers and initiate HCV treatment in non-stigmatizing community setting.

**Methods:**

Between December 2023 and March 2025, persons receiving primary whole person health care on an MPC which offered free rapid HCV testing. Those with positive results were offered on-site confirmatory testing and treatment. Clinical data were collected to assess access, acceptability, and outcomes.

**Results:**

Of the 578 persons who engaged in care on the MPC, 53% were male, 43% Hispanic, 47% identified as White, had a median age of 49 years, 44% had unstable or no housing, and 20% have an opioid use disorder. HCV infection was previously diagnosed in 59 (10.2%), and for those without a previous diagnosis, 32.9% (n=171) accepted rapid HCV testing which identified 5 (2.9%) new infections. Of the 64 with HCV, 2 (3.0%) had recently been tested and found to have an undetectable viral load (VL), and 42.2% (n=27) underwent VL testing, with 11 (40.7%) being found to have an undetectable VL.

Of the 16 with detectable VL, 6 (37.5%) did not initiate treatment (3 were referred to a specialist for cirrhosis evaluation, 1 is pending a follow-up appointment to start treatment, and 2 were lost to follow-up). Ten were initiated on DAA treatment, with1 still receiving treatment and 9 have completed treatment. Of the 9 that having completed treatment: 2 have achieved 12-week SVR, 4 are awaiting 12-week SVR testing, and 3 have completed treatments but were unable to get 12-week SVR confirmation due to transfer of services or being lost to follow-up.

**Conclusion:**

A mobile healthcare model can improve HCV testing and treatment outcomes for vulnerable persons at a community level.

**Disclosures:**

Sheela Shenoi, MD MPH, Merck and Company, Inc.: My spouse worked for Merck 1997-2007 and retains stock in his retirement account. There is no conflict of interest, but included for full disclosure.

